# Ultrasound Imaging of the Periodontium Complex: A Reliability Study

**DOI:** 10.1155/2023/5494429

**Published:** 2023-02-16

**Authors:** Carlos Alberto Figueredo, Lawrence H. Le, Kim Cuong Nguyen, Thanh-Giang La, Edmond H. M. Lou, Neelambar R. Kaipatur, Hollis Lai, Monica P. Gibson, Carlos Flores-Mir, Paul W. Major, Fabiana T. Almeida

**Affiliations:** ^1^School of Dentistry, Faculty of Medicine and Dentistry, University of Alberta, Edmonton, AB, Canada; ^2^Department of Radiology and Diagnostic Imaging, University of Alberta, Edmonton, AB, Canada; ^3^Department of Biomedical Engineering, University of Alberta, Edmonton, AB, Canada; ^4^Department of Electrical and Computer Engineering, University of Alberta, Edmonton, AB, Canada

## Abstract

**Background:**

Ultrasonography is a noninvasive, low-cost diagnostic tool widely used in medicine. Recent studies have demonstrated that ultrasound imaging might have the potential to be used intraorally to assess periodontal biomarkers.

**Objectives:**

To evaluate the reliability of interlandmark distance measurements on intraoral ultrasound images of the periodontal tissues.

**Materials and Methods:**

Sixty-four patients from the graduate periodontics (*n* = 33) and orthodontics (*n* = 31) clinics were recruited. A 20 MHz handheld intraoral ultrasound transducer was used to scan maxillary and mandibular incisors, canines, and premolars. Distances between the alveolar bone crest and cementoenamel junction (ABC-CEJ), gingival thickness (GT), and alveolar bone thickness (ABT) were measured by 3 raters. The intercorrelation coefficient (ICC) and mean absolute deviation (MAD) were calculated among and between the raters. Raters also scored images according to quality.

**Results:**

The ICC scores for intrarater reliability were 0.940 (0.932–0.947), 0.953 (0.945–0.961), and 0.859 (0.841–0.876) for ABC-CEJ, GT, and ABT, respectively. The intrarater MAD values were 0.023 (±0.019) mm, 0.014 (±0.005) mm, and 0.005 (±0.003) mm, respectively. The ICC scores for interrater reliability were 0.872 (95% CI: 0.836–0.901), 0.958 (95% CI: 0.946–0.968), and 0.836 (95% CI: 0.789–0.873) for ABC-CEJ, GT, and ABT, respectively. The interrater MAD values were 0.063 (±0.029) mm, 0.023 (±0.018) mm, and 0.027 (±0.012) mm, respectively.

**Conclusions:**

The present study showed the high reliability of ultrasound in both intrarater and interrater assessments. Results suggest there might be a potential use of intraoral ultrasound to assess periodontium.

## 1. Introduction

The tooth-supporting complex, also known as the periodontium, is formed by the alveolar bone, cementum, periodontal ligament, and gingival tissues [1]. Assessment of the alveolar bone level is vital in the diagnosis, treatment planning, and determining the prognosis of periodontitis and orthodontic treatments [2, 3]. Moreover, the alveolar bone thickness and gingival thickness are important periodontal status to follow during periodontal plastic surgery management, including soft tissue graft and periodontal flap surgery [4].

Chronic inflammation of the periodontium, or periodontitis, is a disease that affects up to 45% of the United States adult population [5]. Periodontitis could lead to alveolar bone destruction and, ultimately, tooth loss [6]. The most important clinical parameter to diagnose periodontitis is the measurement of clinical attachment level/loss (CAL) [6]. The CAL is the distance from the cementum-enamel junction (CEJ) to the bottom of the gingival sulcus. This measurement is routinely done with periodontal probing, a relatively invasive method that includes inserting a probe in the gingival sulcus [7]. However, periodontal probing is unable to assess alveolar bone height and width. Moreover, the CEJ might be hard to detect using a periodontal probe as it usually requires tactile accuracy [8].

Orthodontic treatment can inadvertently result in teeth being moved beyond their alveolar housing, which may result in increased chances of bone loss (dehiscence) and gingival recession. Alveolar bone and gingival thickness assessment (gingival biotype) are important in planning orthodontic treatment and monitoring during treatment to avoid iatrogenic and irreversible tissue loss [2, 9]. Gingival biotype assessment methods include visual assessment, probe insertion, and transgingival probing, which can leave room for interpretation error [10, 11]. Radiographic methods such as 2D radiography and cone-beam computed tomography (CBCT) have been used to image the alveolar bone [12]. However, 2D radiographic methods such as periapical and bitewing radiographs can only assess interdental alveolar bone. The superimposition of bone, gingiva, and root structures heavily limits a reliable visualization of the labial/buccal and lingual/palatal alveolar bone and gingival structures [13]. CBCT provides 3D imaging of the alveolar structures without superimposition, but it might underestimate or overestimate alveolar bone loss [14]. Furthermore, visualization of thin bone tissues such as the alveolar crest requires high image spatial resolution, which requires a much higher radiation dose than conventional 2D radiographs [15, 16]. Therefore, multiple scans to assess alveolar bone and evaluate disease progression and treatment outcomes can result in a questionable cumulative radiation dose over time [15, 16].

Ultrasound (US) is a noninvasive and nonionizing imaging method widely used in medicine and engineering [17–20]. It uses a high-frequency source pulse that echoes and is detected by a transducer [17]. In medicine, US has been widely used to image soft tissues, and in recent years it has been introduced also to image hard tissues such as bone [21, 22]. Ultrasound imaging has become a valuable tool in oral and maxillofacial imaging in recent years, providing high-quality images of oral soft tissues. One specific application of ultrasound is doppler ultrasonography, which allows for the evaluation of blood flow in implant sites, the monitoring of healing in soft tissue grafts, and the detection of oral pathologies [23–27]. This imaging modality is low cost, portable, provides real-time imaging, is comfortable for the patient, and could potentially be used in dental settings to image the labial/buccal alveolar bone. Recent *ex vivo* and clinical studies have reinforced the potential use of US imaging for periodontal assessment, particularly the CEJ, alveolar bone, and gingiva [28–35].

One of the limitations of ultrasound in dentistry is the lack of transducers designed for intraoral use. The mouth anatomy presents spatial limitations for properly manipulating the transducer for maximal diagnostic capability. However, advances in technology have contributed to the development of more miniature multiarray transducers, which can facilitate dental applications. Therefore, the objective of the present study was to investigate the intrarater and interrater reliability of distance measurements between relevant landmarks in the periodontium anatomy using a handheld high-frequency intraoral ultrasound system.

## 2. Materials and Methods

### 2.1. Sample and Data Collection

Sixty-four patients between 10 and 80 years of age (15 males and 49 females) were recruited from the Graduate Periodontics (*n* = 33) and Orthodontics (*n* = 31) Clinics at the Kaye Edmonton Dental Clinic, University of Alberta, between January and August 2021. Any patient possessing natural teeth and older than 10 years of age was considered. This study had ethics approval from the University of Alberta (Pro00099721) and written consent from patients and their parents/guardians.

A customized in-house handheld intraoral ultrasound system was used for imaging. The ultrasound transducer used a 20 MHz imaging frequency, a 7 mm scanning depth, and a default gain of 50%. It was connected to a battery that lasted for up to 45 minutes of continuous scanning. The transducer supported real-time image acquisition with B-mode scanning. Images were transmitted to the Clarius Scanner app (Clarius Mobile Health, BC, Canada) on an iPad Pro (Apple, CA, USA) connected to the transducer via Bluetooth. The iPad transmitted the scans to the imaging application on a Lenovo Legion 5 laptop (Lenovo, Quarry Bay, Hong Kong), which was able to record and save the scans for postassessment.

**Figure 1:**
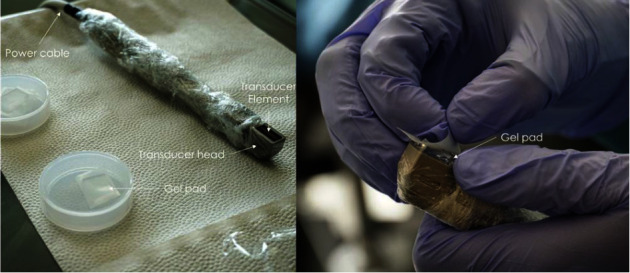
Transducer and gel pad.

US scanning was performed by the first author, CAF (hereby described as R1), a general dentist with ultrasound scanning training provided by an ultrasound expert. Periodontal areas around sixteen teeth were typically scanned in each patient, including the upper and lower incisors, canines, and premolars. Teeth were scanned with the transducer placed buccally, with the long axis of the transducer aligned as closely as possible to the tooth's long axis. A custom-made gel pad was used as an interface between the transducer and tooth to guarantee acoustic coupling to the examined area.  Figure 1 illustrates the transducer and gel pad. The scanning time was around 1 minute per tooth. A total of 1,024 tooth scans in DICOM format were retrieved. Each DICOM video was composed of up to 1000 frames. An optimal frame from each video sequence was selected for linear measurements.

**Figure 2:**
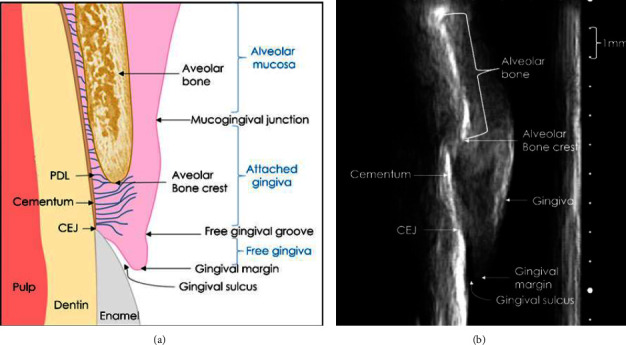
The anatomy of the periodontium compared to an ultrasound image of tooth 11. The (a) was taken from Chan et al. [37] with modification.

**Figure 3:**
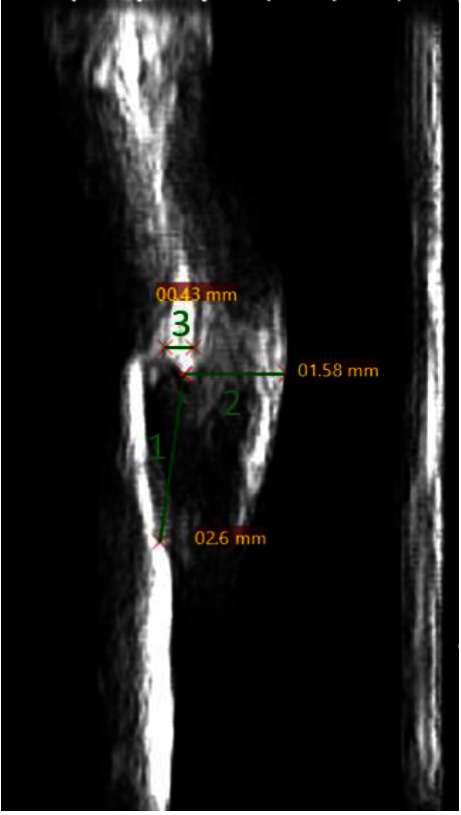
Measurements compared. 1, ABC-CEJ; 2, GT; 3, ABT. Followed by examples of measurements on the same image.

A total of 752 images were used for intrareliability purposes. To assess the reliability between the raters, a sample size calculation based on a previous study using *α* = 0.05, *β* = 0.20, and *π* = 0.3 [36] was done. A sample size of *n* = 180 teeth was used. The images used were selected by the principal investigator to be the most representative of the total data, including all groups of teeth and clinical backgrounds. The file names were coded to blind the raters. Three measurements were conducted for intrarater and interrater reliability. The definitions of the outcome measurements were as follows: alveolar bone crest to CEJ (ABC-CEJ): a straight line from the alveolar bone crest (ABC) to the CEJ; gingival thickness (GT): a straight line from the ABC to the edge of the gingival tissue; alveolar bone thickness (ABT): thickness of the alveolar bone (measured 0.3 mm apical to the alveolar bone crest).  Figure 2 illustrates the periodontium landmarks in an ultrasound image.  Figure 3 illustrates the interperiodontium landmark measurements. All raters conducted measurements on the same images.

### 2.2. Image Assessment and Statistical Analysis

The raters included the first author (R1), an oral and maxillofacial radiologist (R2), and a periodontist (R3). Raters R2 and R3 were calibrated by R1 over two sessions, which consisted of presenting the relevant anatomical landmarks in ultrasound images, the software to be used, and the distances to be measured. Intrarater measurements were performed three times (T1, T2, and T3) by the R1, with two-month intervals between T1, T2, and T3. Raters R2 and R3 measurements were compared to R1 (interrater reliability).

Measurements were conducted in the DenSonics Image Viewer using selected frames from DICOM files. The same frames were used by all observers. ICC with a 95% confidence interval (CI) for intrarater and interrater results was calculated with IBM SPSS (IBM, NY, USA). The types of ICC selected and the classification of scores followed a guideline for reliability research [38]. For intrarater reliability, the ICC was for absolute agreement, and the reported value was for a single measurement. In interrater reliability, the ICC was for consistency, and the reported value was for the average of measurements. A score between 0 and 0.5 was considered poor reliability, between 0.5 and 0.75 was moderate reliability, between 0.75 and 0.9 was good reliability, and between 0.9 and 1 was excellent reliability [38]. SPSS was also used to calculate the means and standard deviations (SDs) of the measurements, as well as the mean absolute deviation (MAD).

Raters were also asked to assess their confidence in identifying the periodontal landmarks based on the image quality score. Image scores were defined as 3: all landmarks clearly seen, high confidence with labelling; 2: one landmark could not be clearly seen, indicating mild confidence with labelling; 1: more than one landmark could not be clearly seen, indicating low confidence with labelling.

## 3. Results

### 3.1. Intrarater Results

From the total 1,024 scans, 752 (73.4%) were used in intrarater reliability, and the rest were excluded for various reasons: missing teeth (26.2%), overlapped teeth (22.6%), tooth fractures (27.8%), bracket interference (5.6%), or inadequate image quality (17.8%). Patient demographics are described in Table 1.

#### 3.1.1. ABC-CEJ

The mean distance for all measures at T1 was 2.81 mm (SD 1.19), T2 was 2.85 mm (SD 1.14), and T3 was 2.88 mm (SD 1.13). The MAD was 0.023 mm (SD 0.02). The ICC for ABC-CEJ T1, T2, and T3 comparison of all teeth was excellent reliability (0.94 (95% CI, 0.93–0.95)). Results for each tooth group are described in Table 2.

In the clinic groups, the means were 2.32 mm (SD 0.84), 2.49 mm (SD 0.85), and 2.43 mm (SD 0.83) for the orthodontics group, and 3.33 mm (SD 1.28), 3.306 mm (SD 1.23), and 3.360 mm (SD 1.29) for the periodontics group. The ICC was good (0.88 (95% CI, 0.86–0.90)) and had excellent reliability (0.95 (95% CI, 0.94–0.96)), respectively.

#### 3.1.2. GT

The mean distance for all measures in T1 was 0.94 mm (SD 0.33), T2 was 0.98 mm (SD 0.33), and T3 was 0.97 mm (SD 0.34). The MAD was 0.01 mm (SD 0.005) The ICC for the GT T1, T2, and T3 comparison of all teeth was excellent reliability (0.95 (95% CI, 0.94–0.96)). Results for each tooth group are described in Table 3.

The means were 0.95 mm (SD 0.34), 0.99 mm (SD 0.34), and 0.99 mm (SD 0.35) for the orthodontics group, and 0.94 mm (SD 0.33), 0.96 mm (SD 0.33), and 0.96 mm (SD 0.33) for the periodontics group. The ICC had excellent reliability (0.95 (95% CI, 0.93–0.96) and 0.96 (95% CI, 0.95–0.97), respectively).

#### 3.1.3. ABT

The mean distance for all measures in T1 was 0.28 mm (SD 0.08), T2 was 0.27 mm (SD 0.08), and T3 was 0.29 mm (SD 0.1). The MAD was 0.005 mm (SD 0.003). The ICC for the ABT T1, T2, and T3 comparison of all teeth was good reliability (0.86 (95% CI, 0.84–0.88)). Results for each tooth group are described in Table 4.

The means were 0.27 mm (SD 0.08), 0.27 mm (SD 0.07), and 0.28 mm (SD 0.07) for the orthodontics group, and 0.28 mm (SD 0.08), 0.28 mm (SD 0.08), and 0.29 mm (SD 0.08) for the periodontics group. The ICC had good reliability (0.84 (95% CI, 0.81–0.87) and 0.87 (95% CI, 0.85–0.89), respectively).

### 3.2. Interrater Results

A total of 180 images were used in each type of measurement of interrater reliability. There were 86 and 94 measurements in the orthodontics and periodontics groups, respectively.

#### 3.2.1. ABC-CEJ

The ICC between the 3 raters was good reliability (0.87 (95% CI, 0.84–0.90)). The mean distance for R1 was 2.1 mm (SD 0.99) For R2 was 2.76 mm (SD 0.91), and for R3 was 2.89 mm (SD 0.87). The MAD was 0.06 mm (SD 0.03). Results for each tooth group are described in Table 5.

In the clinic groups, the means were 2.6 mm (SD 0.85), 2.6 mm (SD 0.83), and 2.71 mm (SD 0.88) for the orthodontics group, and 3.18 mm (SD 1.04), 2.92 mm (SD 0.96), and 3.05 mm (SD 0.82) for the periodontics group. The ICC had good reliability (0.87 (95% CI, 0.82–0.91) and 0.86 (95% CI, 0.80–0.90), respectively).

#### 3.2.2. GT

The ICC between the 3 raters was 0.96 (95% CI, 0.95–0.97). The mean measurement for R1 was 0.86 (0.26) For R2 was 0.83 (0.25), and for R3 was 0.90 (0.23). The MAD was 0.02 mm (SD 0.02). Results for each tooth group are described in Table 6.

In the clinic groups, the means were 0.86 mm (SD 0.25), 0.85 mm (SD 0.24), and 0.91 mm (SD 0.24) for the orthodontics group, and 0.86 mm (SD 0.27), 0.81 mm (SD 0.26), and 0.89 mm (SD 0.23) for the periodontics group. The ICC had excellent reliability (0.98 (95% CI, 0.97–0.98) and 0.94 (95% CI, 0.92–0.96), respectively).

#### 3.2.3. ABT

The ICC between the 3 raters was good reliability (0.84 (95% CI, 0.79–0.87)). The mean measurement for R1 was 0.29 (0.08). For R2 was 0.28 (1.00), and for R3 was 0.35 (0.09). The MAD was 0.03 mm (SD 0.01). Results for each tooth group are described in Table 7.

In the clinic groups, the means were 0.27 mm (SD 0.07), 0.27 mm (SD 0.08), and 0.34 mm (SD 0.08) for the orthodontics group, and 0.30 mm (SD 0.09), 0.29 mm (SD 0.10), and 0.35 mm (SD 0.10) for the periodontics group. The ICC had good reliability (0.83 (95% CI, 0.75–0.88) and 0.84 (95% CI, 0.77–0.87), respectively).

### 3.3. Image Quality Assessment

R1 assessed 752 images and assigned scores ranging from 1 to 5%, 2 to 39%, and 3 to 56%. R2 and R3 assessed 180 images. R2 gave scores ranging from 1 to 24%, 2 to 58%, and 3 to 18%. R3 gave scores ranging from 1 to 43%, 2 to 49%, and 3 to 8%. From the 180 images scored by R2 and R3, R1 scored all of them as 3. Quality assessment results are described in Table 8.

## 4. Discussion

The current study explored the intrareliability and interreliability of measured distances of periodontal landmarks in images taken with an intraoral ultrasound transducer. Results from the present study showed high reliability for all measurements for both intrarater and interrater assessments. The absolute agreement is the appropriate definition type for intrarater ICC, as we assessed how close to the exact measurement the rater was at different assessments [38]. Consistency was selected as the appropriate definition type of interrater ICC, as we intended to assess whether different raters were consistent with each other. With the highest ICC score being gingival thickness in both intrarater and interrater reliability, this represents excellent reliability. The ABC-CEJ score was the second highest; this might be due to a slight disagreement between examiners in identifying the CEJ. The CEJ is an important static periodontal landmark to determine epithelium attachment and bone levels [39]. Difficulty in identifying the CEJ in ultrasound images has been reported in the literature previously [40]. Such a challenge might be due to the different types of contact between cementum and enamel. CEJ identification is also the biggest challenge in periodontal probing, as it is usually subgingival and identified by tactile sensation [8]. Methods of computer-assisted identification of the CEJ in ultrasound images are currently being researched and might facilitate this task in the future [40, 41]. Between the investigated measurements, alveolar bone thickness had the lowest ICC score; however, it still represented good reliability. This might have been due to a slight disagreement in identifying the alveolar bone boundaries in the images. However, the MAD shows that such a difference between measurements by the raters was too small to be clinically significant (0.027 mm).

In this study, crestal bone level results showed good interrater reliability. Assessment of the alveolar bone level is an important factor to understand the level of destruction from periodontal disease and track bone loss during orthodontic tooth movement [9, 42]. This assessment is challenging clinically as the alveolar bone is covered by soft tissues of the periodontium. Direct visualization through a periodontal flap is too invasive and associated with adverse risks. Therefore, a reliable landmark that does not change with age is critical as a reference point in ultrasound images. As the CEJ position does not change regardless of periodontal status, it is used as a landmark to determine hard and soft tissue attachment [43–45].

High-resolution CBCT is the only imaging method used clinically that allows imaging of buccal and alveolar bone levels. US compared to direct measurement was found to be more reliable as compared to CBCT with direct measurement in measuring ABC-CEJ distance. The same authors reported that the US was better at identifying thin alveolar bone than CBCT [33]. This can be attributed to the higher spatial resolution achieved with US images as compared to CBCT. The implementation of ultrasound could potentially reduce patients' exposure to ionizing radiation and reduce its cost. The gingival thickness results showed the highest reliability among the investigated measurements. This comes as no surprise as the US has been used in medicine for soft tissue evaluation with great accuracy. Recent studies have investigated the use of US imaging in assessing gingival thickness [34, 46, 47]. Such assessment is important during implant planning, as research has shown that thicker gingival biotypes have more esthetic results than thin biotypes [48, 49]. Research has also shown that thick gingival types are less likely to suffer from gingival recession during orthodontic tooth movement and following tooth extractions [50, 51]. Currently available methods of measuring gingival thickness include invasive methods such as probe insertion or needle insertion after local anesthesia or noninvasive visual assessments, which are unreliable [11, 34]. There have also been attempts to use CBCT to assess soft tissues; however, this modality has poor soft tissue contrast, which leads to poor accuracy [52]. US has been found to be reliable when compared to direct tissue assessment in edentulous patients [46]. Majzoub et al. recently conducted a study to assess the reliability of ultrasound measurements of soft tissue thickness, soft tissue height, and crestal bone thickness. The study involved 13 raters evaluating ultrasound images, and the results showed good agreement among the raters, indicating that ultrasound is a reliable method for measuring these parameters in the oral and maxillofacial region [47]. The current study supports the findings of Majzoub et al. [47]. Specifically, our study focused on the examination of alveolar bone level and included a larger and more diverse patient population, including individuals with crowding, gingival recession, and bone loss. This allowed for a more comprehensive evaluation of different clinical scenarios and a more representative sample of the general population.

Different tooth groups showed variations in ICC scores based on the position of the tooth in the dental arch. Teeth with a more pronounced dental arch position, such as the canines, were overall easier to scan. This could be attributed to better adaptation of the transducer to the tissues. A comparison of treatment groups showed mean distances for ABC-CEJ in periodontics patients were greater than in orthodontic patients (*p* < 0.001), suggesting that ultrasound imaging was able to accurately identify and measure bone loss in periodontitis patients. MAD results showed that despite some differences between means that were statistically significant, the clinical difference in millimeters was minimal. For comparison, a periodontal probing has a 1 mm margin of error, and the interrater MAD was 0.63 mm [53].

The assessment of the clarity of images in identifying periodontal landmarks between R1, R2, and R3 showed varied results. US scanning and data collection by R1 attributed to the familiarity in clearly identifying periodontal landmarks compared to other raters. R1 scored most images as 3 (56%); R2 and R3 scored most images as 2 (58% and 49%, respectively). R3 had the highest percentage with a score of 1 (43%), and the lowest percentage with a score of 3 (8%). It is important to note that R1 was the most familiar with US images, followed by R2, who is an imaging expert and has previous experience reading ultrasound images. R3 was the only rater who had experience interpreting ultrasound images during the calibration process. This should be taken into account while interpreting results as each rater had a different level of experience with US. It can also be noted that scores for anterior teeth are overall higher than posterior images; we hypothesize that this could be due to tooth anatomy. Recent literature has reported the importance of training in interpreting US images [47]. Survey results from Majzoub et al. [47] and results from our study show that there might be importance in implementing educational tools that can help train dental professionals in reading US. Incorporating US teaching in dental schools as one of the diagnostic methods for assessing the periodontium can be a future task for dental educators.

The strengths of the present study include the large number of images assessed for intrarater reliability. Also, images were derived from patients from both orthodontics and periodontics clinics, with different periodontal conditions and ages ranging from 10 to 80 years of age. The sample included patients with gingival recession, enamel erosion, alveolar bone loss, braces, and resin-based attachments. The variety in the sample group shows the potential application of ultrasound in scenarios ranging from healthy periodontium to patients with periodontitis. This study, along with recently published data [33, 34, 47], provide support that the US has the potential to become a noninvasive diagnostic tool for both soft and hard tissues in dental clinics.

The present study was limited by the fact that the transducer head was too large to scan second premolars and molars. The design of the transducer was also not conducive to scanning the anatomy of the palatal and lingual surfaces of teeth. Orthodontic patients with brackets or attachments were also challenging to scan. Moreover, at the current stage, the consistency of the scan quality and sample image selection is heavily operator-dependent. This study used a transducer prototype, and future models may have the potential to overcome these limitations. Finally, it is important to mention that, to date, there has been a lack of consistency across the literature regarding ideal ultrasound frequencies for investigating oral mucosa. The next step in this research would be to determine the accuracy of ultrasound by comparing it to the gold standard of direct measurements. For example, the distance between ABC-CEJ could be compared between ultrasound images and direct measurements taken during periodontal flap surgery. Other studies could investigate the potential use of computer-assisted localization of the periodontium landmarks as an educational tool to assist dental practitioners who are new to ultrasound imaging.

## 5. Conclusion

This study showed high reliability to evaluate a subset of periodontium anatomical structures from selected US imaging in patients with different clinical periodontal situations by raters from different clinical backgrounds and years of experience. Results suggest that there might be potential for implementing ultrasound in routine dentistry as a noninvasive tool to assist in diagnosis.

## Figures and Tables

**Table 1:** Patient demographics.

Patient demographics (*n* = 64)	Values
Total age statistics (mean; std. deviation; range)	33.6; ±19.2; 12–77
Periodontics *n*/orthodontics *n*(%)	33/31(51%/49%)
Female *n*/male *n*	49/15(76%/24%)

Periodontics patient demographics (*n*=33)	Values
Total age statistics (mean; std. deviation; range)	46.7; ±17.1; 14 − 77
Female *n*/male *n*	24/9 (73%/27%)

Orthodontics patient demographics (*n*=33)	Values
Total age statistics (mean; std. deviation; range)	19.8; 8.7; 12–45
Female *n*/male *n*	25/6 (81%/19%)

**Table 2:** Intrarater ABC-CEJ by tooth groups and clinic groups.

Group	*N*	ICC (95% CI)	T1 means (Std. dev)	T2 means (Std. dev)	T3 means (Std. dev)
Total	752	0.940 (0.932–0.947)	2.812 (±1.190)	2.847 (±1.140)	2.880 (±1.135)
Upper incisors	205	0.940 (0.925–0.952)	2.779 (±0.977)	2.766 (±0.969)	2.803 (±0.960)
Upper canines	109	0.973 (0.962–0.981)	3.279 (±1.368)	3.159 (±1.245)	3.212 (±1.228)
Upper premolars	86	0.945 (0.923–0.962)	3.235 (±1.309)	3.227 (±1.279)	3.208 (±1.247)
Lower incisors	183	0.922 (0.900–0.939)	2.613 (±1.160)	2.709 (±1.143)	2.756 (±1.155)
Lower canines	88	0.892 (0.840–0.927)	2.594 (±1.086)	2.803 (±1.091)	2.831 (±1.107)
Lower premolars	81	0.949 (0.928–0.966)	2.502 (±1.203)	2.587 (±1.13)	2.612 (±1.136)
Orthodontics	389	0.878 (0.856–0.897)	2.323 (±0.844)	2.418 (±0.851)	2.431 (±0.831)
Periodontics	363	0.953 (0.944–0.960)	3.335 (±1.283)	3.306 (±1.228)	3.360 (±1.218)

**Table 3:** Intrarater GT by tooth groups and clinic groups.

Group	*N*	ICC (95% CI)	T1 means (Std. dev)	T2 means (Std. dev)	T3 means (Std. dev)
Total	752	0.953 (0.945–0.961)	0.943(±0.335)	0.977 (±0.334)	0.974(±0.341)
Upper incisors	207	0.944 (0.926–0.958)	1.203 (±0.335)	1.246 (±0.356)	1.249 (±0.371)
Upper canines	107	0.939 (0.913–0.957)	0.918 (±0.284)	0.959 (±0.257)	0.945 (±0.265)
Upper premolars	87	0.951 (0.931–0.966)	0.996 (±0.253)	1.008 (±0.259)	0.1.01 (±0.257)
Lower incisors	185	0.930 (0.909–0.947)	0.792 (±0.266)	0.830 (±0.266)	0.822 (±0.268)
Lower canines	88	0.933 (0.905–0.954)	0.724 (±0.221)	0.751 (±0.226)	0.751 (±0.219)
Lower premolars	78	0.837 (0.774–0.887)	0.835 (±0.214)	0.859 (±0.194)	0.853 (±0.195)
Orthodontics	390	0.948 (0.935–0.958)	0.950 (±0.345)	0.989 (±0.341)	0.989 (±0.353)
Periodontics	362	0.960 (0.951–0.967)	0.936 (±0.326)	0.964 (±0.326)	0.958 (±0.328)

**Table 4:** Intrarater ABT by tooth groups and clinic groups.

Group	*N*	ICC (95% CI)	T1 means (Std. dev)	T2 means (Std. dev)	T3 means (Std. dev)
Total	752	0.859 (0.841–0.876)	0.278 (±0.081)	0.275 (±0.081)	0.289 (±0.101)
Upper incisors	211	0.882 (0.852–0.906)	0.284 (±0.084)	0.283 (±0.089)	0.296 (±0.088)
Upper canines	108	0.878 (0.937–0.911)	0.271 (±0.084)	0.272 (±0.078)	0.278 (±0.075)
Upper premolars	90	0.764 (0.685–0.829)	0.299 (±0.067)	0.294 (±0.075)	0.298 (±0.067)
Lower incisors	180	0.828 (0.783–0.865)	0.268 (±0.074)	0.264 (±0.073)	0.278 (±0.069)
Lower canines	85	0.891 (0.848–0.924)	0.263 (±0.091)	0.263 (±0.084)	0.273 (±0.081)
Lower premolars	78	0.855 (0.793–0.901)	0.282 (±0.077)	0.275 (±0.78)	0.292 (±0.073)
Orthodontics	387	0.842 (0.814–0.866)	0.272 (±0.078)	0.269 (±0.077)	0.280 (±0.075)
Periodontics	365	0.874 (0.851–0.895)	0.283 (±0.084)	0.282 (±0.085)	0.293 (±0.080)

**Table 5:** Interrater ABC-CEJ by tooth groups and clinic groups.

Group	*N*	ICC (95% CI)	T1 means (Std. dev)	T2 means (Std. dev)	T3 means (Std. dev)
Total	180	0.872 (0.836–0.901)	2.911 (±0.993)	2.756 (±0.915)	2.887 (±0.867)
Upper incisors	30	0.780 (0.597–0.888)	2.692 (±1.056)	2.201 (±0.949)	2.770 (±0.991)
Upper canines	30	0.855 (0.733–0.926)	3.061 (±1.215)	2.872 (±1.145)	3.057 (±1.040)
Upper premolars	30	0.873 (0.768–0.935)	3.108 (±0.853)	2.951 (±0.691)	2.816 (±0.744)
Lower incisors	30	0.794 (0.623–0.895)	2.974 (±0.807)	3.094 (±0.606)	3.030 (±0.585)
Lower canines	30	0.963 (0.932–0.981)	3.049 (±0.882)	3.122 (±0.786)	3.070 (±0.846)
Lower premolars	30	0.928 (0.869–0.963)	2.583 (±1.045)	2.299 (±0.810)	2.579 (±0.871)
Orthodontics	86	0.874 (0.820–0.914)	2.618 (±0.854)	2.579 (±0.834)	2.713 (±0.884)
Periodontics	94	0.859 (0.801–0.902)	3.179 (±1.039)	2.919 (±0.959)	3.046 (±0.823)

**Table 6:** Interrater GT by tooth groups and clinic groups.

Group	*N*	ICC (95% CI)	T1 means (Std. dev)	T2 means (Std. dev)	T3 means (Std. dev)
Total	180	0.958 (0.946–0.968)	0.861 (±0.263)	0.830 (±0.253)	0.899 (±0.235)
Upper incisors	30	0.973 (0.950–0.986)	1.133 (±0.322)	1.100 (±0.255)	1.094 (±0.265)
Upper canines	30	0.806 (0.644–0.901)	0.833 (±0.188)	0.7510 (±0.251)	0.891 (±0.215)
Upper premolars	30	0.963 (0.933–0.981)	0.963 (±0.215)	0.938 (±0.179)	1.035 (±0.168)
Lower incisors	30	0.967 (0.939–0.983)	0.745 (±0.186)	0.737 (±0.100)	0.772 (±0.190)
Lower canines	30	0.970 (0.945–0.985)	0.705 (±0.228)	0.720 (±0.235)	0.781 (±0.217)
Lower premolars	30	0.955 (0.918–0.977)	0.787 (±0.156)	0.733 (±0.139)	0.825 (±0.134)
Orthodontics	86	0.978 (0.968–0.985)	0.862 (±0.255)	0.855 (±0.244)	0.906 (±0.239)
Periodontics	94	0.941 (0.918–0.959)	0.860 (±0.272)	0.807 (±0.261)	0.894 (±0.232)

**Table 7:** Interrater ABT by tooth groups and clinic groups.

Group	*N*	ICC (95% CI)	T1 means (Std. dev)	T2 means (Std. dev)	T3 means (Std. dev)
Total	180	0.836 (0.789–0.873)	0.287 (±0.083)	0.280 (±1.000)	0.346 (±0.090)
Upper incisors	30	0.792 (0.618–0.894)	0.285 (±0.086)	0.268 (±0.072)	0.359 (±0.133)
Upper canines	30	0.737 (0.517–0.866)	0.266 (±0.079)	0.242 (±0.069)	0.331 (±0.054)
Upper premolars	30	0.912 (0.838–0.955)	0.323 (±0.087)	0.325 (±0.098)	0.385 (±0.091)
Lower incisors	30	0.844 (0.713–0.920)	0.275 (±0.078)	0.257 (±0.100)	0.333 (±0.077)
Lower canines	30	0.935 (0.698–0.916)	0.272 (±0.77)	0.271 (±0.098)	0.344 (±0.081)
Lower premolars	30	0.827 (0.683–0.912)	0.303 (±0.080)	0.315 (±0.093)	0.328 (±0.080)
Orthodontics	86	0.826 (0.751–0.881)	0.274 (±0.069)	0.267 (±0.085)	0.338 (±0.080)
Periodontics	94	0.837 (0.770–0.886)	0.299 (±0.092)	0.291 (±1.099)	0.354 (±0.099)

**Table 8:** Image quality scores by raters and groups. Number of images with each score and percentage from the total.

Tooth group	Score	R1	R2	R3
Total	1	39 (5%)	44 (24%)	78 (43%)
	2	294 (39%)	104 (58%)	89 (49%)
	3	419 (56%)	32 (18%)	13 (8%)

Upper incisors	1	9 (4%)	1 (3%)	9 (30%)
	2	71 (34%)	17 (57%)	13 (43%)
	3	125 (62%)	12 (40%)	8 (27%)

Upper canines	1	6 (5%)	0	6 (20%)
	2	43 (39%)	20 (67%)	22 (73%)
	3	60 (56%)	10 (33%)	2 (7%)

Upper premolars	1	4 (5%)	5 (17%)	12 (40%)
	2	49 (57%)	22 (73%)	17 (57%)
	3	33 (38%)	3 (10%)	1 (3%)

Lower incisors	1	9 (5%)	9 (30%)	13 (43%)
	2	65 (35%)	17 (57%)	16 (54%)
	3	109 (60%)	4 (13%)	1 (3%)

Lower canines	1	6 (7%)	14 (47%)	18 (60%)
	2	28 (31%)	15 (50%)	12 (40%)
	3	54 (62%)	1 (3%)	0

Lower premolars	1	5 (6%)	15 (50%)	20 (67%)
	2	38 (47%)	13 (43%)	9 (30%)
	3	38 (47%)	2 (7%)	1 (3%)

## Data Availability

The data used to support the findings of this study are restricted in order to protect patient privacy.
